# Factors prompting and deterring suicides on the roads

**DOI:** 10.1192/bjo.2023.52

**Published:** 2023-05-03

**Authors:** Hilary Norman, Lisa Marzano, Rachel Winter, Ioana Crivatu, Jay-Marie Mackenzie, Ian Marsh

**Affiliations:** Faculty of Social Sciences and Humanities, Middlesex University, London, UK; Faculty of Science and Technology, Middlesex University, London, UK; Psychology Department, University of Westminster, UK; Faculty of Medicine, Health and Social Care, Canterbury Christ Church University, UK

**Keywords:** Suicide, roads, self-harm, impulsive, suicide method

## Abstract

**Background:**

In addition to the devastating impact on the individual and their families, suicides on the roads can cause distress and harm to other people who might be involved in a collision or witness an attempt. Despite an increased focus on the characteristics and circumstances of road-related suicides, little is known about why people choose to end their lives in this way.

**Aims:**

The aim of the current study was to investigate the factors prompting and deterring the decision to attempt suicide on the roads.

**Method:**

We conducted a secondary analysis of survey data, as well as seven in-depth qualitative interviews. Participants had lived experience of suicidal ideation or behaviour at a bridge or road location. We also carried out an online ethnography to explore interactions in different online communities relating to this method of suicide.

**Results:**

Participants perceived a road-related suicide to be quick, lethal, easy and accessible and to have the potential to appear accidental. The proportion of participants who described their thoughts and attempts as impulsive appeared to be higher than had been observed with other method choices. The potential impact on other people was a strongly dissuasive factor.

**Conclusions:**

Measures designed to prevent access to potentially lethal sites may be particularly important, given that many participants described their thoughts and behaviour as impulsive. In addition, fostering a culture of care and consideration for other road users may help to dissuade people from taking action on the roads.

Road-related suicide methods include jumping off or on to road infrastructure, stepping into the path of a moving vehicle, or driving off or into road infrastructure or into another vehicle.^1^ It has been estimated that at least 50 people take their lives on UK roads each year.^1^ However, the exact number of suicides on the roads is difficult to establish with certainty, as road-related incidents may be misclassified as traffic accidents in official records and national statistics.^2^ In Sweden, a re-examination of all road traffic fatalities to take into account psychosocial information about the individual, as well as the circumstances of the death, resulted in a significant increase in the proportion classified as suicides.^3^

Suicidal behaviour on the roads has a lasting impact, not only for the individual concerned and their family and friends, but also for other road users who might be injured or involved in a collision, or who might witness the incident.^4^ Indeed, one study in Switzerland found that significantly more people were killed as a consequence of road-related suicide than with other suicide methods.^5^ There is also a wider impact on the road network as a whole, with fatal and non-fatal incidents leading to costly road closures and delays.^1^

Previous studies have investigated the characteristics of people who go to the roads to end their lives. The majority of individuals are male,^5–7^ in common with suicide deaths in the general population. A study into non-fatal self-harm on the roads found that the most common road-related method among men was crashing a vehicle, whereas the most common method among women was jumping from a bridge or walking out in front of a vehicle.^6^ People who use a road-related method of suicide tend to be younger compared with all suicide deaths.^6^ Studies of people who have thought about or survived suicide attempts on the roads suggest that the intent to die is high^8^ and higher than in relation to other methods.^6^

Despite the increasing interest in road-related suicides, little is known about why people might choose this particular method to end their life. One study about the factors influencing choice of suicide method found that among people who attempted suicide by putting themselves in front of a vehicle, method effectiveness was cited most frequently.^9^ This appears to be the case particularly in relation to suicides on the railways, where the perceptions of lethality tend to be overestimated.^10^ However, it is not known whether the same perceptions are held in relation to road-related methods. Furthermore, it may be that the various types of road suicide are associated with different persuasive and dissuasive factors. Studying the method-specific factors that influence suicidal thoughts can result in important insights for preventive interventions.^10^ A recent Cochrane review on interventions to prevent road-related suicides highlighted the lack of existing evidence and specifically recommended further research into the reasons people might come to the road network to end their life.^11^

The aim of the current study was to investigate further the factors prompting and deterring the decision to attempt suicide on the roads.

## Method

The research question was explored using a multi-methodological approach. First, we carried out a secondary analysis of a data-set presented in two previously published studies,^9,10^ focusing specifically on responses relating to road-related suicidal ideation and behaviour. Second, we conducted seven in-depth qualitative interviews with people who had experience of feeling suicidal at a bridge or road location. Finally, we carried out an online ethnography to explore interactions in different online communities relating to this method of suicide.

### Ethics

The authors assert that all procedures contributing to this work comply with the ethical standards of the relevant national and institutional committees on human experimentation and with the Helsinki Declaration of 1975, as revised in 2008. All procedures involving human subjects/patients were approved by the Psychology Department Research Ethics Committee at Middlesex University (reference: ST019-2015 and 7045-2021).

### Online survey

The anonymous survey was hosted on Qualtrics, and was open to participants between July 2015 and 2016. It was advertised through suicide prevention organisations such as Samaritans UK, online forums, social media and special interest groups. Study posters and leaflets were also placed on university bulletin boards, at local branch offices of relevant charities and in the National Suicide Prevention Alliance newsletter and were mailed out to supporters of the charity Campaign Against Living Miserably (CALM). No exclusion criteria were applied. Participants did not receive any remuneration.

Participants gave their informed consent by clicking a box. Participants were asked whether they had ever experienced suicidal thoughts. If so, they were asked to describe any particular methods they had contemplated and the reasons for these, and whether they had suggestions for suicide prevention at the specific locations they had considered. In many cases, this prompted participants to comment on perceived barriers to the use of those methods and/or locations. Similar questions were asked in relation to suicide attempts. All questions allowed open-ended, free-text responses with no word limit or prompts.

#### Analysis

The data-set for the current study consisted of those survey responses that concerned suicidal thoughts and actions at or relating to road or bridge locations. Open-ended survey data were analysed inductively for content^12^ by one researcher (I.C.), according to a coding book agreed by three members of the research team.

Survey data are presented as frequencies or percentages, as appropriate. Coded data were used to calculate descriptive statistics, with denominators varying in relation to individual variables because of missing information.

### Interviews

#### Participants and recruitment

We recruited an opportunity sample of participants for interview from among those who had taken part in a second online survey in 2019.^13^ Participants were aged 18 years or over and had stated in the survey that they had felt suicidal at a road or bridge location. We contacted 41 people meeting these criteria who had not previously been interviewed for a different follow-up study. Nine people responded positively and seven people agreed to be interviewed. Participants were offered £20 renumeration.

#### Procedure and interviews

All potential participants received briefing information in advance of the interview, which outlined the purpose of the study and contained details of relevant support organisations. Oral (recorded) consent was obtained at the start. Evidence suggests that participating in research about suicide or self-harm does not lead to a significant increase in distress or the urge to self-harm.^14^ However, given the sensitivity of the subject, steps were taken to minimise any possible impact on participants. The interviewer and the participant completed a personalised safety plan together. The interviewer was a trained Samaritan listening volunteer (H.N.). A visual analogue scale^15^ was used at the beginning and end of the interview to assess the impact on mood.

#### Procedure and materials

Semi-structured interviews were carried out in January and February 2021 over Zoom video conferencing. Participants were asked to describe incidents in their lives where they had felt suicidal at a road or bridge location, or where they had experienced suicidal thoughts relating to roads or bridges (e.g. ‘Could you tell me, in your own way, about your [most recent] experience of attempting to end your life on the roads?’). Prompting questions were used to explore the background to the experiences, including the degree of preparation or premeditation, the circumstances and the feelings relating to the incident itself (or the ideation), reasons for and thoughts about the choice of method relative to other methods, and participants’ views on prevention. The interviews ranged in duration from 44 to 73 min (average 63 min). They were audio recorded and transcribed verbatim.

#### Analysis

The transcripts were analysed using thematic analysis.^16^ One researcher (H.N.) coded all the transcripts inductively, line by line, using NVIVO software. Two transcripts were independently coded by J.-M.M., and emerging themes were discussed among members of the research team (L.M., J.-M.M. and H.N.).

### Online ethnography

Online ethnography was used to gather an in-depth understanding of how people were engaging with and discussing suicide on the roads in online spaces.^17^

Two researchers (R.W. and I.M.) explored suicide forums, Reddit and Twitter to provide a range of voices. First, key terms related to road and suicide were searched for on each site. As the research progressed, and through continual analysis of the data, we noted further terms that were being used to talk about suicide on the road. This cyclical method was an effective way to explore conversations relating to jumping or walking into traffic and suicide by car accident. We particularly analysed the ensuing conversations that happened in the comments associated with the original posts.

Data were collected from April 2018 to December 2020. In total, we analysed 2306 original posts and 4169 comments posted on suicide forums, Reddit and Twitter. Reflecting offline ethnographic data collection, field notes were kept throughout the data collection to capture our observations. To respect the online communities we researched, and in an endeavour to reduce traceability of this population, we have not included direct quotes, user names, or the terms/hashtags that were used to find the data.

#### Analysis

Analysis was conducted throughout the data collection and once the final data-set has been compiled. Thematic analysis was then used to analyse the field notes and posts.^16^ Codes and developing findings were continually discussed within the research team (I.M. and R.W.) to help increase the validity of the analysis.

### Integrating the analyses

The three strands of the study were conducted separately and analysed independently. Subsequently, the research team reviewed and discussed the findings in the light of two main research questions.

- Why do people choose a road or bridge location to end their lives?
- Why do they reject a road or bridge location?

Convergent themes were identified. Notable divergent findings were discussed and are also presented here, where they deepen our understanding of road suicides.

## Results

Of the 1398 people who took part in the online survey, 340 reported having considered (*N* = 323/1398, 23.1%) and/or attempted (*N* = 50/712, 7.0%) suicide at a road or bridge location (Table 1). More than half the participants (173/318, 54.4%) were under the age of 35 years (including 78 participants who were aged between 18 and 25 years), and two-thirds were female (209/320, 65.3% *v*. 102 males, 31.9%; six participants described themselves as nonbinary and three as transgender males).

**Table 1:** Characteristics of survey participants

	Road suicide ideation (*N* = 323)		Road suicide attempts (*N* = 50)	
	*n*/*N*	%	*n*/*N*	%
Female (compared with men and other gender)	200/303	66.0%	29/50	58.0%
Median age (minimum to maximum)	32	16–69	32	16–70
White ethnicity (versus other ethnic groups)	266/323	82.4%	44/50	80.0%

Causing a collision while driving	125/323	38.7%	11/50	22%
Stepping in front of a moving vehicle	106/323	32.8%	21/50	42.0%
Jumping off or on to road infrastructure	130/323	40.2%	19/50	38.0%

Seven interviewees, five women and two men, were aged between 28 and 58 years (Table 2). All had experienced suicidal thoughts at a road location; none had taken action on the roads resulting in injury to themselves or others. Five participants had attempted to take their own lives using a different method (usually overdose).

**Table 2:** Characteristics of interviewees

	Age	Gender	Employment	Method of suicidal behaviour or ideation
P1	53	Female	Employed	Driving/bridge
P2	31	Male	Student/self-employed	Bridge
P3	56	Female	Employed	Bridge/stepping out in front of vehicle
P4	58	Male	Employed	Bridge
P5	50	Female	Employed	Stepping out in front of vehicle
P6	28	Female	Employed	Bridge/stepping out in front of vehicle
P7	29	Female	Student	Stepping out in front of vehicle

### Persuasive factors

**Fig. 1:**
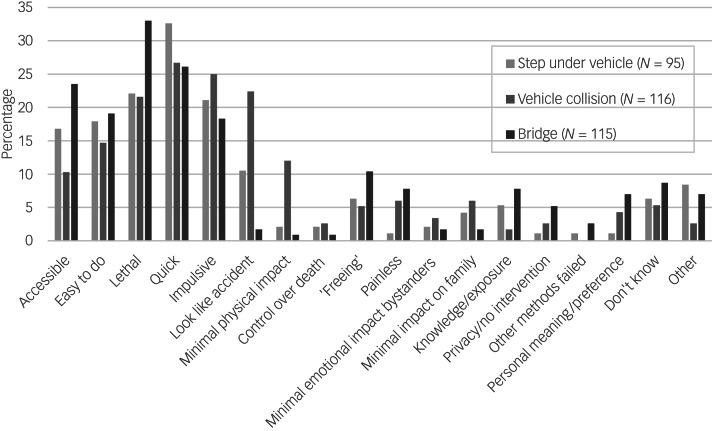
Self-reported reasons for suicidal thoughts about a road or bridge location (*N* = 323).

**Fig. 2:**
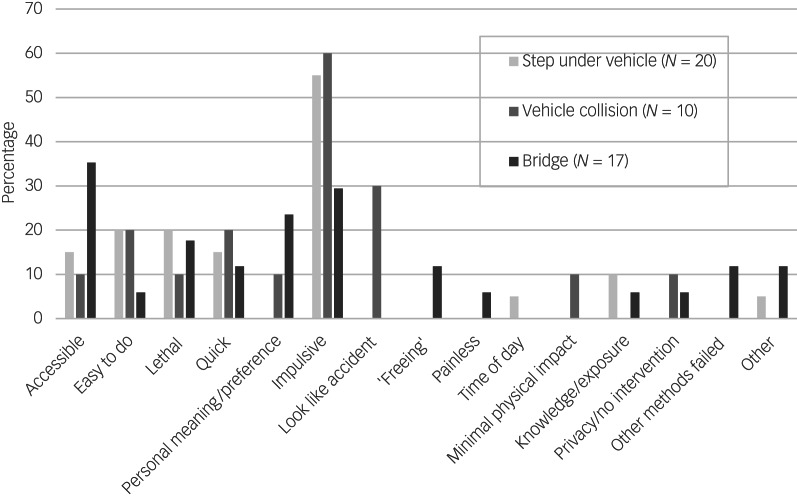
Self-reported reasons for suicide attempts at a road or bridge location (*N* = 50).

Survey participants’ self-reported reasons for thinking about and attempting suicide using a road-related method are illustrated in Figs. 1 and 2, respectively. Common persuasive factors were identified across the survey, interview and ethnographic analyses, although they differed in some cases according to the method considered (e.g. jumping from a bridge, stepping out in front of a vehicle or causing a collision) and according to whether people were describing suicidal thoughts or suicide attempts. The reasons given for considering a specific road-related method were often multiple: in the survey, 48.3% of those who commented on their motivations (*N* = 143) mentioned at least two factors.

#### Quick

The survey responses indicated that road suicide was perceived as a quick method (Fig. 1). Although this was mentioned in relation to all types of road suicide method, it was particularly the case for stepping in front of a vehicle, for which it was the most commonly cited factor in relation to suicidal thoughts.

‘*I knew it would be quick*’ (Survey participant, SP)

Similarly, online, people commented on posted videos of people being hit or run over by a vehicle, saying that it looked like a fast method to die. Four interview participants also thought that suicide on the roads would be quick, giving little time to reflect or regret, particularly in contrast to other methods such as taking an overdose.

#### Lethal

Road-related methods were also perceived as effective means of taking one's life. Of the three methods, jumping from a bridge in particular was perceived as highly lethal.

*‘I thought the fall would be fatal*.’ (SP)

Interview participants who had considered jumping from a bridge expressed the view that there were several opportunities for fatal injury within the one act.

‘*So thinking the idea was like I would time it if I didn't, the fall wouldn't kill me, then a car or a van ideally a van or something would finish the job*.’ (Interview participant, IP)

On all of the online platforms, when people initially posted that they were thinking about dying on the roads, they would describe it as a quick or reliable method. They had seen videos and news stories shared online, where someone had died by suicide on the road, which appeared to have created the perception that this was a lethal method. However, the reliability of road-related suicide was often challenged by other community members.

#### Easy and accessible

The accessibility of the road network was a strong persuasive factor to emerge from the survey (Fig. 1) and interviews.

‘*Quick, easy/very little planning involved*’ (SP)

Among the survey participants who had attempted suicide, accessibility appeared to be an important factor for those who had jumped from a bridge (Fig. 2).

‘*It's right outside my house and took minimal effort*’ (SP)

A related theme to emerge from the interviews was the ubiquity of road and bridge locations in daily life. If participants were to come across potential places when feeling upset or suicidal, it might cause them to view such locations differently.

‘*Firstly it was I was I was just walking, to walk, to yeah to um get anywhere, get just you know walk [laughs] never mind where um and almost sort of unintentionally I ended up at that place* [road location]. *The thought process being what it was it was like … oh look just look what you could do with this!’* (IP)

In the online ethnography, road-related methods were perceived as more accessible than other methods. Some people explained that suicide on the roads or by car was their last resort. They spoke about not being able to purchase substances or equipment required for other suicide methods but had a car or a road nearby where they could die by suicide. Some wrote that they thought about jumping from a bridge, walking in front of a vehicle or crashing their car every day, especially when they were on or by a road.

#### Appearing accidental…

Thoughts about dying by causing a collision in one's vehicle were particularly associated across all research strands with wanting the death to appear accidental. This was for two main reasons. First, on the suicide forums and subreddits relating to suicide and mental health, some people were concerned that if their death were deemed a suicide, their relatives would not be eligible for insurance money. Second, people spoke about not wanting to cause distress to their families. They thought that dying by suicide in a way which looked like an accident might carry less stigma. This sentiment was echoed by a third of the survey participants who had attempted suicide by causing a collision.

‘*It wouldn't necessarily be seen as suicide. I wanted to avoid the stigma for my two children.’* (SP)

#### …or leaving it to fate and other people

An interesting angle on this theme is that some participants themselves did not want to take the final decision to die. The idea of death occurring as a result of an ‘accident’, even one that occurred as a result of their own behaviour, was appealing.

‘*And I think probably you know, in the hope that I would crash the car and I wouldn't have to think about it, I almost like that the whole decision would be taken away from me because I would I would hit something and… And then my fate would be decided.’* (IP)

Online, particularly on Twitter, people wrote that they wanted to die but could not or did not want to take their own life, and therefore they wanted someone else to hit them with a car. Unlike many other methods, road-related methods appeared to offer the chance of dying without having to take the final action oneself.

#### Impulsive

Forty-four per cent of survey participants who had attempted to end their lives on the roads described their suicide attempts as impulsive, opportunistic or without prior reflection, particularly with regards to attempts involving a road collision (60%) or stepping in the path of a vehicle (55%, Fig. 2).

‘*Turning my bike into the path of a car on an A road … I saw an opportunity and didn't think about making the decision.’* (SP)

Thoughts relating to suicide on the roads were also described as impulsive for 25% of participants who had thought about causing a collision and 21% of people who had thought about stepping out in front of a vehicle (Fig. 1).

Similarly, five of the seven interview participants used words like instinct, impulse, urge, spontaneous or opportunistic to describe their suicidal ideation on roads or bridges.

‘*At times my mood is quite bad, I can go out for a walk and there's just that, I'm next to a road and there's that I could just do that and that quite strong urge.’* (IP)

Although words like ‘spontaneous’, ‘urge’ or ‘opportunity’ were used to describe the thoughts or actions associated with specific suicidal crises, it should be noted that three of the seven interview participants had lived with suicidal thoughts for many years. For these participants, urges experienced in a specific situation were set against a backdrop of chronic suicidality and planning. One participant described how they would walk from their house when they felt suicidal and, in doing so, would come across potential locations for suicide. The interviewer asked whether they would describe this behaviour as premediated or spontaneous.

‘*I spend my entire life, or have spent my entire life, planning and looking and … so you ask me a method and I could probably tell you about it. But the actual impulse to do … well it's not an im … is it an impulse? Not quite sure what it is, but the compulsion to do so is uncontrollable.’* (IP)

### Dissuasive factors

Of the 323 survey respondents who contemplated suicide on the roads, just under half (159, 49.2%) also reported a suicide attempt (as well as past suicidal ideation). In most cases (126/159, 79.2%), this did not involve a road-related method. Forty individuals who had contemplated a road-related method but had attempted suicide in a different way commented on the reasons they did not attempt to end their lives on the roads. Two main reasons were given: the impact on others and the risk of surviving with injuries.

#### Impact on ‘innocent’ others

The most cited dissuasive factor among survey participants (17/40 who gave reasons) was the impact on other people, particularly drivers and other road users. This was also the main reason given by interview participants.

‘*As I'm standing on a road bridge and there's all those cars coming on or vans or lorries or whatever I jump out for I'm affecting that person's life too. And, through no fault, and I said I didn't– that it wasn't the friends or the family that snapped me out of it, it was the idea I was getting involved an innocent, and I didn't want to do that.’* (IP)

The impact on other people was a strong theme on the online suicide forums. In response to posts about dying by causing a collision in a car, many users tried to dissuade the individual from choosing this suicide method because of the impact on the other driver, including loss of license and job, long-term trauma and mental health conditions. Community members commented that suicide should be an individual act and not involve others. They said that it was selfish if others were involved, and that innocent people may be affected, such as passengers in the other car or the people who had to clean the scene in the aftermath of a suicide.

#### Surviving with injuries

Another dissuasive factor observed online was the risk of surviving with injuries. In some cases, people shared their own stories about living with physical health problems, having, for example, tried to end their life by jumping from a bridge. With regard to causing a collision or stepping out in front of a vehicle, people online felt there were many variables that were hard to control and which might result in injury rather than death, such as the behaviour of other road users, the speed of the car and the vehicle's safety features.

Twelve of 40 survey participants who gave reasons they did not choose a road method for suicide cited the risk of surviving with injuries.

‘*I may just become paralysed*’ (SP)

However, in the in-depth interviews, the possibility of surviving with life-changing injuries was only mentioned by one person and, at the time, they had not seen this as a deterrent. Instead, even injury was perceived to be a means of changing the situation they were in.

‘*My pain would go away because either I would be dead or I would be so much physical pain that I didn't have to think about the emotional pain I was experiencing.’* (IP)

## Discussion

To our knowledge, this is the first study to explore the persuasive and dissuasive factors that might influence a person's decision to end their life on the roads. Across the three research strands (survey, interview, online) we found that people held perceptions that this would be a quick, lethal and accessible death that could in some circumstances be interpreted as an accident. In addition, thoughts and particularly behaviours relating to suicide on the roads were frequently described as impulsive. The strongest dissuasive factors were the impact on other people and the risk of surviving with injuries.

Although there were similarities in the reasons given for choosing or rejecting each of the three main road-related methods (stepping out in front of a vehicle, causing a collision or jumping from a bridge), there were also marked differences. For example, the wish to disguise the suicide as an accident related particularly to car collisions. Jumping from a bridge was perceived to be a highly lethal method. These differences suggest that road-related methods should be treated separately for purposes of risk assessment and prevention.

A notable finding from the survey was the high proportion of participants who described their road-related suicidal behaviour as impulsive (44% across all three method types). The current sample was drawn from a wider survey examining thoughts and actions relating to all suicide methods.^9^ This wider survey found that across all methods, only 12% of participants who had attempted suicide described their actions as impulsive. The interviews suggest that the ubiquity of the roads in everyday life may play a part in making road suicide more impulsive than other methods. The fact that participants described their experiences at a road or bridge location as, to some extent, impulsive or opportunistic underlines the importance of measures to delay or prevent action being taken at the scene.

What constitutes an ‘impulsive’ act is contested in the literature, and different studies have used different measures, including degree of preparation, duration of preceding suicidal ideation and, as in the current study, self-report.^18^ These measures do not appear to be closely related to each other. May and Klonsky (2015) found that self-reported impulsive motivation for a suicide attempt was not related to the degree of preparation and only moderately related to contemplation of suicide for more than 3 h prior to the attempt.^19^ Similarly, the interviews in the current study indicate how, even where the thoughts or the actions were described as impulsive, they may still have occurred in the context of pre-existing suicidal ideation and even planning and research. Suicidal acts have been associated with heightened impulsivity in the context of negative emotional states.^20^ It may be, therefore, that someone with existing suicidal ideation may experience impulsive suicidal thoughts or behaviours when feeling distressed and confronted with the opportunity for suicide on the roads.

The scale of the road network makes this an accessible method of suicide, in comparison with other methods that might require more planning or preparation. Access to means is a strong influencing factor in choice of suicide method and underlies some of the observed different national trends.^21^ However, despite the relative ease of access to cars and roads, road-related suicide methods remain relatively rare in the UK.^22^ This suggests that the dissuasive factors identified in the current study may play an important part in an individual's choice of method. In particular, the study identified the potential impact that a suicide can have on other people as a strongly dissuasive factor. The same phenomenon was identified in a study on the factors prompting and deterring the decision to end one's life on the railways.^10^ Interestingly, we observed how this argument was used actively online to dissuade people from using this method. Sociocultural acceptability has been proposed as a contributory factor in the choice of suicide method, explaining, for example, gender differences such as the greater use of firearms by men.^23^

### Implications

The findings outlined in this study suggest some measures that could be helpful in reducing the number of people who attempt suicide on the roads, which are discussed below. Although they are focused on one specific method, the evidence is that restricting recourse to one means of suicide does not result in displacement to a different method^24^ and can therefore lead to a general reduction in suicides.

The findings of the current study suggest a need for preventive measures that may restrict, delay or disrupt impulsive behaviour on the road network. Although physical barriers are costly and difficult to put in place everywhere, their use could help to address impulsive suicidal urges at particular road or bridge locations known to be high risk. An analysis of 13 observational before-and-after studies across six countries found that measures to restrict access to means of suicide by jumping saw suicides reduced by 91%.^25^ Other ‘softer’ and potentially less expensive measures designed to disrupt suicidal thought processes such as signs, lighting or ‘pseudo-barriers’ (e.g. lasers or motion sensors) have been piloted in other settings, such as at railway locations.^26^ However, the evidence for the effectiveness of such measures is currently more limited.^27–30^

Online, we saw how people appeared to be dissuaded from a road-related method by community members who stressed the potential lethal impact on other people. Although this may be effective in a closed, peer-led discussion, it is a difficult message to employ more widely because of the risk of appearing to dismiss or stigmatise the distress of the suicidal individual.^31,32^ In a more general way, fostering a sense of community, care and responsibility towards other road users might motivate individuals to consider the wider impact of their behaviour. The revision to the UK Highway Code may present an opportunity to promote such a message.^33^ The new Code advises that all road users should be ‘considerate to other road users and understand their responsibility for the safety of others’ (Rule H1).

Future research in this area should focus on the differences in the factors that prompt and deter use of each road-related method to inform targeted interventions.

### Limitations

The strength of the current study lies in the use of different methods, which increases confidence in the triangulated findings. In addition, participants were recruited from different environments. For example, recruitment for the lived experience survey and interviews relied largely on tweets and adverts featuring official organisations such as Samaritans with a strong emphasis on suicide prevention. By contrast, some online data were gathered from ‘pro-choice’ spaces which are explicitly critical of suicide prevention (‘pro-life’) approaches. Nonetheless, the views and experiences of these different groups do not necessarily reflect those of others who have considered or attempted to take their own lives on the roads.

The number of interviews conducted was relatively small although consistent with other mixed-method studies of this type.^34^ In addition, there was heterogeneity among the interview participants in terms of the type of road-related suicide method considered or attempted.

All the data analysed for this study were from people who have had thoughts about suicide on the roads or who have survived an attempt. The processes and experiences involved in a fatal attempt could in some important ways be different. As with all research on suicide, it is not possible to generalise from one group to another with any certainty.

### Summary of findings

Suicide by a road-related method was perceived to be quick, lethal and accessible. The factors prompting a decision to attempt suicide on the roads varied according to the method considered (jumping from a bridge, stepping out in front of a vehicle, causing a collision). Jumping from a bridge was associated with high lethality, whereas participants considered that stepping in front of a vehicle was likely to be a quick means of dying. The possibility of the death appearing accidental was a persuasive factor for those who thought about causing a collision. Thoughts and particularly attempts involving road-related methods were frequently described as impulsive, suggesting that interventions to prevent access to potentially lethal locations may be particularly important. Across all three research strands, the potential impact on other people was a strongly dissuasive factor. Through an exploration of lived experience relating to the decision to attempt suicide on the roads, these findings add to the emerging body of work on the characteristics and circumstances of road suicides.

## Data Availability

Owing to the sensitive nature of this research, participants of this study were not asked for consent for their data to be made available to others for further research, so supporting data are not available.
